# Molecular Characterization of Carbonic Anhydrase II (CA II) and Its Potential Involvement in Regulating Shell Formation in the Pacific Abalone, *Haliotis discus hannai*

**DOI:** 10.3389/fmolb.2021.669235

**Published:** 2021-05-07

**Authors:** Md. Rajib Sharker, Zahid Parvez Sukhan, Kanij Rukshana Sumi, Sang Ki Choi, Kap Seong Choi, Kang Hee Kho

**Affiliations:** ^1^Department of Fisheries Science, College of Fisheries and Ocean Sciences, Chonnam National University, Yeosu, South Korea; ^2^Department of Fisheries Biology and Genetics, Faculty of Fisheries, Patuakhali Science and Technology University, Patuakhali, Bangladesh; ^3^Department of Aquaculture, Faculty of Fisheries, Patuakhali Science and Technology University, Patuakhali, Bangladesh; ^4^Department of Biological Sciences, College of Life Industry and Science, Sunchon National University, Jeonnam, South Korea; ^5^Department of Food Science and Technology, Sunchon National University, Jeonnam, South Korea

**Keywords:** *Haliotis discus hannai*, carbonic anhydrase, qRT-PCR, ontogenesis, *in situ* hybridization

## Abstract

Carbonic anhydrases (CAs) are a family of metalloenzymes that can catalyze the reversible interconversion of CO_2_/HCO_3_^–^, ubiquitously present in both prokaryotes and eukaryotes. In the present study, a CA II (designated as *HdhCA II*) was sequenced and characterized from the mantle tissue of the Pacific abalone. The complete sequence of *HdhCA II* was 1,169 bp, encoding a polypeptide of 349 amino acids with a NH_2_-terminal signal peptide and a CA architectural domain. The predicted protein shared 98.57% and 68.59% sequence identities with CA II of *Haliotis gigantea* and *Haliotis tuberculata*, respectively. Two putative N-linked glycosylation motifs and two cysteine residues could potentially form intramolecular disulfide bond present in *HdhCA II*. The phylogenetic analysis indicated that *HdhCA II* was placed in a gastropod clade and robustly clustered with CA II of *H. gigantea* and *H. tuberculata*. The highest level of *HdhCA II* mRNA expression was detected in the shell forming mantle tissue. During ontogenesis, the mRNA of *HdhCA II* was detected in all stages, with larval shell formation stage showing the highest expression level. The *in situ* hybridization results detected the *HdhCA II* mRNA expression in the epithelial cells of the dorsal mantle pallial, an area known to express genes involved in the formation of a nacreous layer in the shell. This is the first report of *HdhCA II* in the Pacific abalone, and the results of this study indicate that this gene might play a role in the shell formation of abalone.

## Introduction

Carbonic anhydrases (CAs) are zinc ion-containing metalloenzymes that can catalyze the essential hydration of CO_2_ through the simple chemical reaction: CO_2_ + H_2_O ⇄ HCO_3_^–^ + H^+^ (Lindskog and Silverman, 2000). CAs play an essential role in multiple physiological processes such as pH regulation, electrolyte balance, ionic transportation, carboxylation or decarboxylation reactions, biocalcification, and tumorigenicity (Supuran, 2008, 2011; Alterio et al., 2009). CAs are important components of the CO_2_-concentrating mechanisms in different groups of algae. They could increase the rate of photosynthesis (Qu et al., 2018). In erythrocytes, CA is a superabundant enzyme that plays an indispensable role in CO_2_ transport by catalyzing the dehydration of plasma HCO_3_^–^ ions (Geers and Gros, 2000; Henry and Swenson, 2000; Perry and Gilmour, 2006). The cytosolic CA in gill may contribute to provide counter ions for maintaining pH balance and ionic regulation in fish (Henry and Swenson, 2000; Marshall, 2002). In mollusk, CA seems to be contributed to shell formation *via* catalyzing the hydration of CO_2_ (Nielsen and Frieden, 1972). This enzyme has been shown to be an effective catalyst in the calcification mechanism of coral (Rahman and Oomori, 2010).

Carbonic anhydrase isozymes were isolated in the erythroid cells of mammals and have been subsequently identified in most organisms (Meldrum and Roughton, 1933; Rudenko et al., 2015). Eight evolutionarily distinct families of CAs, including α, β, γ, δ, ζ, η, θ, and ι, have been reported in unicellular and multicellular organisms (Zolfaghari et al., 2020). Their amino acid residues share no significant identities and seem to be evolved independently from distinct inherited genes (Krishnamurthy et al., 2008; Bertucci et al., 2009; Del Prete et al., 2015; Supuran and Capasso, 2015; Kikutani et al., 2016). Among these families, α-CA is widely distributed in animals and plants (Aspatwar et al., 2010). α-CA exhibits the highest catalytic activity in the hydration reaction than β- and η-CA. On the contrary, γ-, δ-, and ζ- CA isozymes possess the lowest enzymatic efficiencies (Capasso and Supuran, 2015; Supuran and Capasso, 2015). In mammals, 16 isoforms of α-CA isozymes have been explored, of which 13 are catalytically effective and 3 are non-catalytic due to the absence of one or more functionally active histidine amino acid residues (Sly and Hu, 1995; Tashian et al., 2000). The functions of each isozyme vary pursuant to their molecular sequences, kinetic attributes, sensitivities to inhibitors, tissue distributions, and subcellular localizations (Hewett-Emmett, 2000; Lehtonen et al., 2004).

Carbonic anhydrase II is a secreted and membrane-bound α-CA that can catalyze carboxylation and decarboxylation reactions. The typical structure of CA II contains three histidine (His) residues that can bind to Zn^2+^ ion, and a proton (H^+^) shuttling residue that is responsible for converting a Zn-bound water molecule to hydroxide ion. In addition, gate-keeping signature residues (namely, Glu-106 and Thr-199 in human CA) allow excellent orientation of Zn-bound hydroxide ion to increase the nucleophilic attack of a substrate (Christianson and Fierke, 1996; Lindskog and Silverman, 2000). CA II not only participates in the hydration reaction but also plays an important role in the osmoregulatory functions of fish (Grosell et al., 2007).

The Pacific abalone is a commercially important molluscan bioresources in China, Japan, and Korean Peninsula. *Haliotis discus hannai* is considered as a popular seafood item worldwide due to its contents of health beneficial bioactive molecules (Suleria et al., 2017). Previous studies have characterized cytosolic CA isozymes in vertebrates and invertebrates (Pongsomboon et al., 2009; Le Roy et al., 2012; Ali et al., 2015; Pan et al., 2016; Sumi et al., 2019). However, the characterization and expression analysis of CA isozymes in Pacific abalone have not yet been reported. In this study, the complete sequence of CA II isozyme was first cloned from the mantle of *H. discus hannai*, and its spatiotemporal expression was determined using the molecular assay.

## Materials and Methods

### Animals and Sample Collection

Three-year-old adult male and female Pacific abalone, *H. discus hannai* (total body mass: 128.2 ± 0.86 g; shell length: 10.5 ± 0.12 cm) were collected from Jindo Island, South Korea and transferred to the laboratory, College of Fisheries and Ocean Science, Chonnam National University (CNU). The tissues from the cerebral ganglion, mantle, gill, heart, shell muscle, hemocyte, testis, and ovary were collected, immediately frozen in liquid nitrogen, and kept at − 80°C for further RNA isolation. All experimental embryonic and larval samples were collected as described previously (Sharker et al., 2020a). The cryosection from the mantle tissue was prepared following the previous protocol (Sharker et al., 2020b, c, d). The experimentation was performed according to the guidelines of the Institutional Animal Care and Use Committee of CNU (approval number: CNU IACUC-YS-2020-5).

### RNA Extraction and cDNA Synthesis

Total RNA was isolated from different tissues of an experimental animal using an RNeasy mini kit (Qiagen, Hilden, Germany) following the kit protocol. The quality of each RNA sample was evaluated using 1% (w/v) agarose gel electrophoresis and quantified by spectrophotometry on a NanoDrop^®^ NP 1000 device (Thermo Fisher Scientific, Waltham, MA, United States). Subsequently, 1 μg of RNA was transformed into cDNA employing Superscript^®^ III cDNA synthesis kit (Invitrogen, Carlsbad, CA, United States) as per the kit instruction.

### Cloning and Sequencing of Full-Length cDNA of CA II

A pair of primer (forward: 5′-GTGGCAGTCTTCCTATCTAC-3′; reverse: 5′-GCTGCATCATCACCTGCCA-3′) was designed based on the nucleotide sequence of *Haliotis gigantea* CA isozyme (GenBank accession no. AB500104.1). Reverse transcription polymerase chain reaction (RT-PCR) amplification reactions were carried out using the following amplification program: 3 min at 95°C, followed by 35 cycles of 2 min at 94°C, 1 min at 58°C, 1 min at 72°C, with a final extension step at 72°C for 5 min. The purification was carried out using the PCR purification kit as per the kit protocol. Subsequently, the purified fragments were cloned into pTOP Blunt V2 vector (Enzynomics, Daejeon, South Korea) and transformed into DH5α-competent *Escherichia coli* cells (Enzynomics). Then Plasmid DNA from selected clones was isolated using plasmid miniprep kit (Qiagen, Hilden, Germany) and sequenced by a sequencing company (Macrogen, South Korea). Complete sequence of CA II was obtained from *H. discus hannai* by rapid amplification of cDNA ends (RACE) using a Smarter^®^ RACE cDNA Kit (Clontech Laboratories, Inc., United States) as per the protocol provided by the manufacturer. The touchdown PCR was carried out with 25 cycles for 3′-RACE and 30 cycles for 5′-RACE using gene-specific primers (GSPs) set (antisense primer: 5′-GATT ACGCCAAGCTTCCATGGCTCCTGTACACGGTTCTTCC-3′, sense primer: 5′-GATTACGCCAAGCTTCACTTTGTCTGAG AGCGTCCTGTGGC-3′), a universal primer mix (UPM), and SeqAmp DNA Polymerase in 50 μL of reaction volume following the instruction provided by the manufacturer. The resultant PCR products were purified, ligated into linearized pRACE vector, transformed into Stellar Competent Cells, and finally sequenced as described earlier.

### Sequence and Phylogenetic Analysis

The nucleotide and amino acid sequence of Pacific abalone CA II was analyzed with BLAST at the NCBI database. A web-based tool “SMART” was used for the prediction of CA domain architecture (Letunic and Bork, 2018). Expert protein analysis system was used to evaluate the physiochemical properties and subcellular localization of this gene (Gasteiger et al., 2003). Multiple sequence alignment was created using Clustal Omega package (Sievers et al., 2011; Alva et al., 2016). The Jalview Java alignment editor was employed to edit and visualize multiple sequence alignment (Waterhouse et al., 2009). Predictions of the N-linked glycosylation sites and serine/threonine phosphorylation sites were performed with NetNGlyc 1.0 server (Chuang et al., 2012) and NetPhosK 3.1 server (Blom et al., 1999), respectively. The N-terminal signal peptide and disulfide bond were predicted using SignalP 4.1 (Petersen et al., 2011) and CYSPRED (Fariselli et al., 1999), respectively. To generate a phylogram, vertebrate and molluscan CAs were curated from NCBI using BLASTP program. A phylogenetic analysis was conducted with MEGA software (version 7.0) using bootstrap analysis for 1,000 replicates (Kumar et al., 2016).

### Template Identification and Three-Dimensional Homology Modeling of *H. discus hannai* CA II

Modeler^1^ was used for the analysis of high-resolution three-dimensional (3D) homology modeling of *H. discus hannai* CA II isozyme by optimally satisfying spatial restraints (Šali and Blundell, 1993). Human CA II 3D structure (1.07 Å) template was considered to generate the 3D model of Pacific abalone CA II. Protein Quality Predictor (Wallner and Elofsson, 2003), Verify3D (Eisenberg et al., 1997), and ERRAT tools were used for assessing the stereochemical quality of the predicted protein model (Colovos and Yeates, 1993). UCSF Chimera program was used for interactive visualization and analysis of the predicted CA II 3D structure (Pettersen et al., 2004).

### Semiquantitative RT-PCR

A primer set (forward: 5′-GAACAGGGTGTGTGACACG-3′ and reverse: 5′-GCAGAACGATGTCCGAAATAG-3′) designed from the cloned sequence was applied to conduct semiquantitative RT-PCR. Ribosomal protein L-5, RPL-5 (GenBank accession no JX002679.1) (forward: 5′-TGTCCGTTTCACCAACAAGG-3′ and reverse: 5′-AGATGGAATCAAGTTTCAATT-3′), was selected as a reference gene based on its expression stability (Wan et al., 2011). The PCR amplification conditions were similar to those described earlier.

### Quantitative RT-PCR Analysis

The quantitative RT-PCR (qRT-PCR) was carried out in triplicates using 2 × qPCRBIO SyGreen Mix Lo-Rox on a LightCycler^®^ 96 System (Roche, Germany) in a 20-μL reaction mixture. Three biological replicates (*N* = 3) were used for each tissue and ontogenetic sample. The same gene-specific and RPL-5 primers used for semiquantitative RT-PCR analysis were used for qRT-PCR. The PCR amplification programs were subjected to a predenaturation step at 95°C for 2 min, followed by 40 cycles of denaturation at 95°C for 1 min, annealing at 60°C for 30 s and 72°C for 1 min. Relative mRNA expression was assessed using the 2^–ΔΔCT^ method.

### Statistical Analysis

Data were statistically analyzed using one-way ANOVA followed by Tukey’s multiple comparisons using SPSS (version 16.0) to assess whether the means were significantly different. Statistically significant difference was set at *p* < 0.05.

### *In situ* Hybridization

Digoxenin (DIG)-labeled RNA antisense and sense probes were synthesized from the CDS region of CA II sequence by *in vitro* transcription as described earlier (Sharker et al., 2020e, f). The hybridized tissue sections of the mantle were incubated with a blocking solution at RT for 1 h and then treated with an antibody at − 20°C overnight. Subsequently, the tissue sections were incubated with a labeling mix and kept in a dark place to attain color. Finally, the slides were examined under a stereomicroscope.

## Results

### Identification and Characterization of CA II From *H. discus hannai*

**FIGURE 1:**
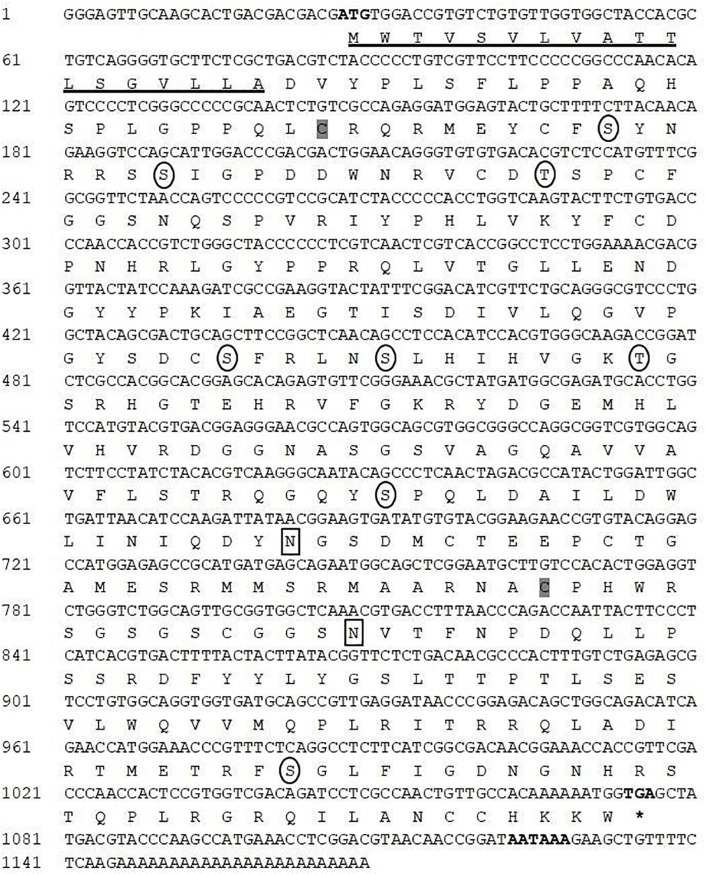
Nucleotide and amino acid sequence of *HdhCA II*. The start codon, stop codon (asterisks), and polyadenylation signal (AATAAA) are marked in bold. The N-terminal signal peptide is underlined. The N-linked glycosylation site is enclosed in a rectangular box. The circles indicate potential phosphorylation sites in the mature protein. Two cysteine residues (Cys-40 and Cys-247) could potentially form intramolecular disulfide bond and are shaded in gray.

The complete cDNA sequence of CA II was isolated and cloned from the mantle tissue of *H. discus hannai* and referred to as *HdhCA II* (GenBank accession number MT876410). Its nucleotide sequence was 1,169 bp in length encoding a polypeptide of 349 amino acids with the calculated molecular mass and isoelectric point (p*I*) of 38.93 kDa and 8.58, respectively (Figure 1). The protein domain analysis revealed that *HdhCA II* (from ^50^Y to ^344^C) showed similarity with a potential CA isoform II. Its coding region comprised a predicted signal peptide (18 amino acids) followed by a cleavage site between Ala^18^ and Asp^19^. The cloned sequence contained two N-linked glycosylation sites and eight phosphorylation sites at positions ^49^S, ^55^S, ^67^T, ^137^S, ^142^S, ^150^T, ^202^S, and ^319^S. Two cysteine residues (Cys-40 and Cys-247) in this sequence are likely to form intramolecular disulfide bond for the enzyme biosynthesis.

The protein BLAST analysis demonstrated that the predicted CA II sequence shared the highest identities with *H. gigantea* and *Haliotis tuberculata* CA II. The alignment of fish and mammalian vertebrate CA sequences revealed that the cloned Pacific abalone CA II sequence shared 29.03%, 28.34%, and 29.67% sequence identities with human (*Homo sapiens*, NP_000058.1), mouse (*Mus musculus*, NP_033931.4), and zebra fish (NP_954685.1, *Danio rerio*) CA II, respectively (Table 1).

**TABLE 1:** Amino acid sequence identities of *HdhCA II* with CA IIs of other gastropod mollusk, eutherian mammals, and piscine vertebrates.

1	2	3	4	5	6	7	8	9	
	81.15	80.77	62.69	62.65	34.91	30.34	29.13	29.03	1. Human
		93.46	60.38	61.09	36.64	30.72	28.99	28.34	2.Mouse
			60.77	60.70	34.91	29.10	28.16	27.74	3.Rat
				72.76	32.33	30.10	30.07	29.67	4. Zebra fish
					32.13	29.97	30.51	29.43	5. Rainbow trout
						31.29	29.72	28.75	6. Sea hare
							69.16	68.59	7. Green ormer
								98.57	8. Giant abalone
									9. Pacific abalone

**FIGURE 2:**
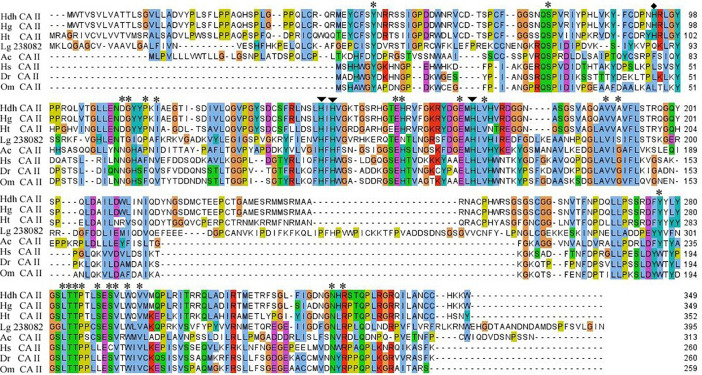
Multiple sequence alignment of *HdhCA II* and CA IIs of other representative invertebrate and vertebrate species. Three zinc ligand histidine residues and proton shuttling residues are indicated by arrows and diamond circle, respectively. Amino acid residue in the catalytic site involved in the hydrogen bond formation is denoted by asterisks. Hdh, *H. discus hannai*; Hg, *H. gigantea*; Ht, *H. tuberculata*; Lg, *Lottia gigantea*; Ac, *Aplysia californica*; Hs, *Homo sapiens*; Dr, *Danio rerio*; and Om, *Oncorhynchus mykiss*.

The *in silico* analysis indicated that this protein might be an extracellular (secreted) protein. The active site amino acid residues in CA domain of Pacific abalone and other cytoplasmic CAs of vertebrates and invertebrates are highly conserved (Figure 2). The three histidine residues predicted to form Zn^2+^ in the active site are also conserved in all CA isoforms. The histidine residue (^94^H) important for proton shuttling is also conserved in molluscan CAs. In addition, several other highly conserved amino acids are found in this cloned sequence.

**FIGURE 3:**
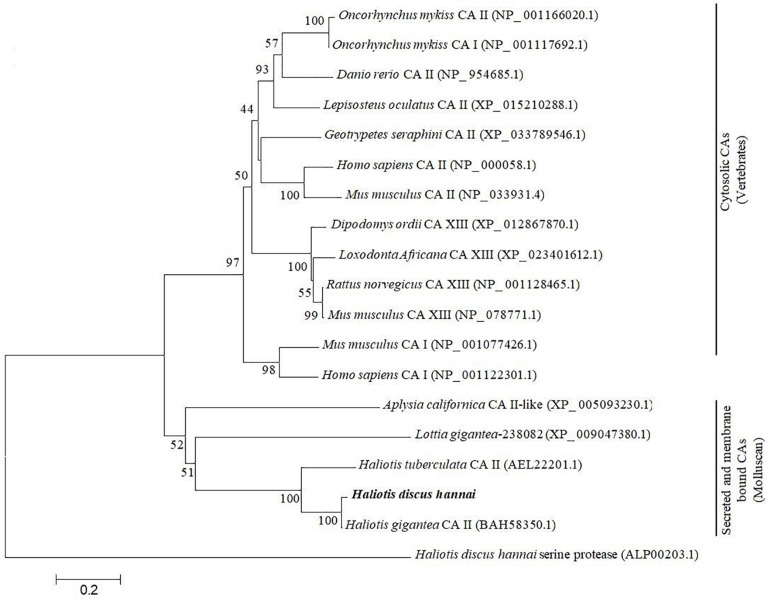
Molecular phylogenetic analysis of carbonic anhydrase isoform was constructed using NJ approach with 1,000 bootstrap replications. The scale bar at the bottom represents the amino acid divergence per site. The numbers in phylogram nodes indicate percentage bootstrap values for the phylogeny. *HdhCA II* is highlighted in bold.

The phylogenetic analysis was performed using CAs of representative species of vertebrates, and molluscan with the neighbor joining (NJ) method to infer evolutionary connections. The phylogenetic tree showed two major clades: (1) cytosolic CAs in vertebrates and (2) secreted and membrane-bound CAs in mollusk. The CA II of *H. discus hannai* was placed in the molluscan clade and phylogenetically clustered with *H. gigantea* CA II with a high bootstrap value (Figure 3).

**FIGURE 4:**
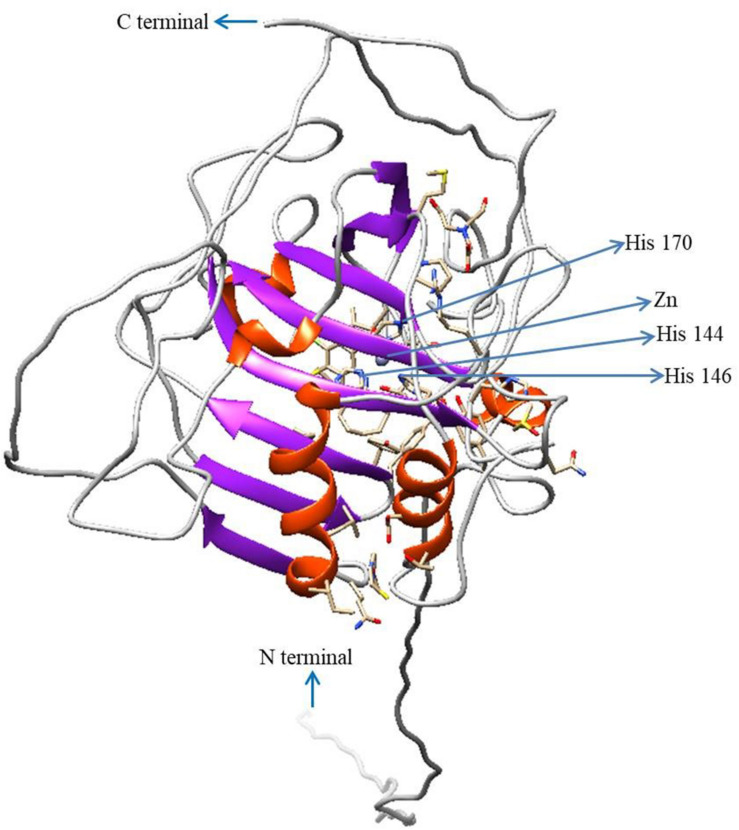
Three-dimensional structure of *HdhCA II*. The N- and C-termini are marked with blue arrows. Zn^2+^ and coordinated three histidine residues are indicated by blue arrows. The structure was generated using UCSF Chimera software.

To predict the 3D model of CA II, the crystal structure of human CA II (PDB 4Q08) was selected based on the high identities of several amino acid signatures (Figure 4). The evaluation results of this predicted model were as follows: ProQ, LG score of 2.780 (value > 1.5 indicates very good model), and MaxSub sore of 0.585 (value > 0.5 indicates a very good model); Verify3D: 3D/1D profile score of 89.34%; and ERRAT quality factor of 92.94%.

### Expression Analysis of *HdhCA II* mRNA

**FIGURE 5:**
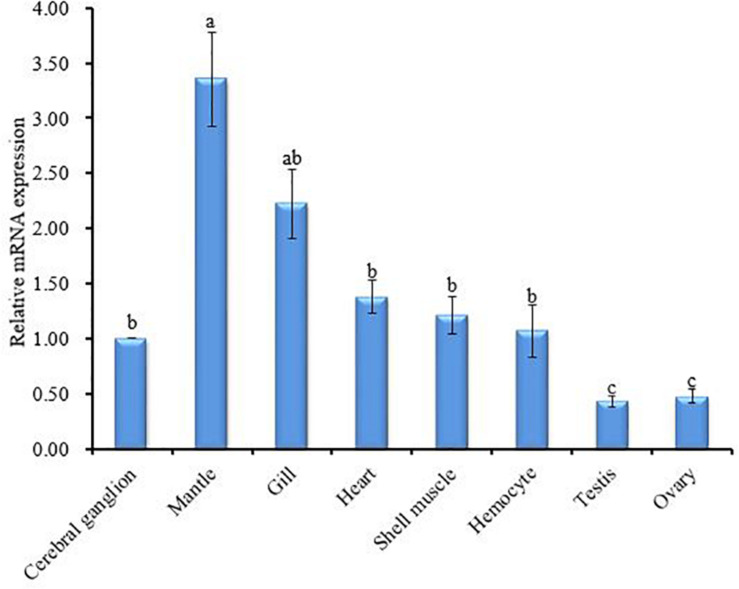
*HdhCA II* mRNA expression (means ± SD, *N* = 3) in different tissues of Pacific abalone is based on quantitative real-time PCR (qRT-PCR). The expression pattern of *HdhCA II* mRNA in all tissues is calibrated by the expression in the cerebral ganglion (1). Different characters in vertical bar indicate significantly (*p* < 0.05) different.

The tissue-specific expression profile of CA II was analyzed by qRT-PCR. The mantle tissue exhibited the highest level of *HdhCA II* mRNA expression than other tested tissues (Figure 5). The expression of *HdhCA II* mRNA among cerebral ganglion, heart, shell muscle, and hemocyte showed no significant differences. A significantly lower expression was found in gonadal tissues (i.e., testis and ovary). The supporting data are shown in Supplementary Figure 1.

**FIGURE 6:**
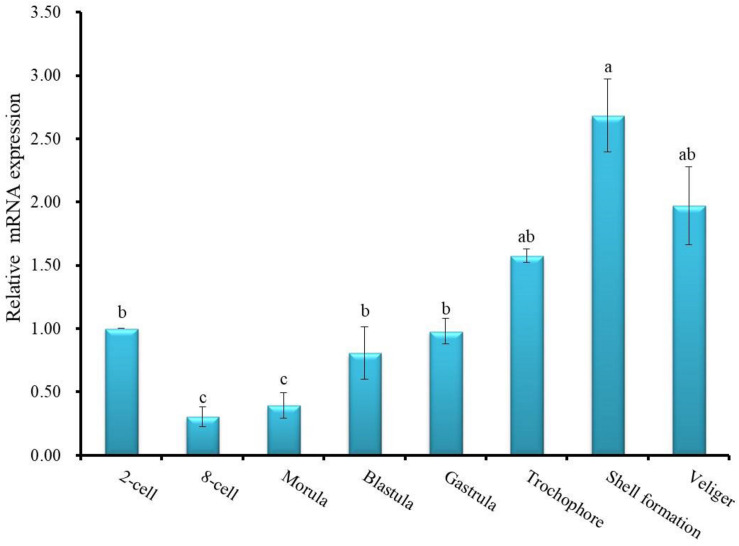
*HdhCA II* mRNA expression (means ± SD, *N* = 3) in different ontogenetic stages of the Pacific abalone. The expression levels of *HdhCA II* mRNA in various stages are calibrated by its expression in the two-cell stage (1). Different letters indicate significantly (*p* < 0.05) different.

To investigate the functional role of *HdhCA II* during ontogenetic development of the Pacific abalone, the expression patterns of CA II mRNA transcript in different stages of development were determined using the qRT-PCR assay. The results of the analysis revealed that *HdhCA II* mRNA was expressed throughout the early developmental stages in a ubiquitous fashion (Figure 6). The *HdhCA II* mRNA levels were relatively low in multicellular stages until gastrula. The expression level was highest in the shell formation stage compared with other examined stages.

**FIGURE 7:**
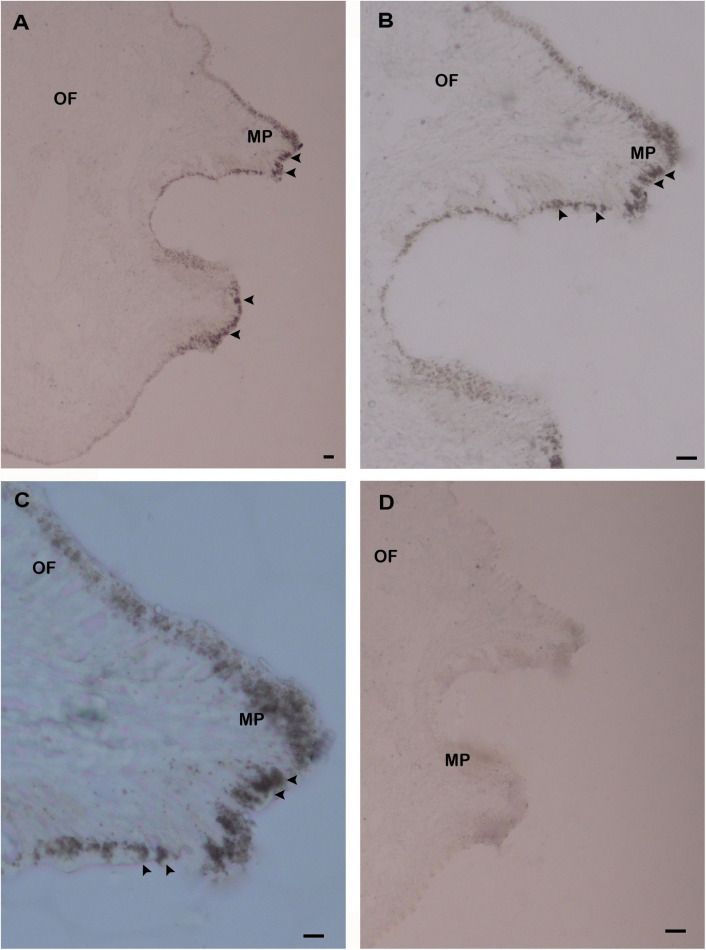
The localization of *HdhCA II* mRNA in the mantle tissue of Pacific abalone detected by ISH. **(A)** The positive hybridization signals were detected in the epithelial cells of the dorsal mantle pallial (MP), **(B)** the medium magnification of A, **(C)** the higher magnification of A, and **(D)** the negative control section showed no hybridization signals. The positive signals are marked with black arrowheads. Scale bar, 100 μm. OF, outer fold; MP, mantle pallial.

The *in situ* hybridization (ISH) was carried out using the mantle tissue sections to elucidate the functional role of *HdhCA II* mRNA in the shell formation of *H. discus hannai*. The *HdhCA II* mRNA hybridized signal was found in epithelial cells of the dorsal mantle pallial, an area known to express genes involved in the nacreous layer synthesis of the shell (Figures 7A–C). However, the negative control (sense probe) showed no hybridization signal (Figure 7D).

## Discussion

Carbonic anhydrases play an important role in many physiological processes by catalyzing the hydration reaction. In mollusks, CAs have been previously identified in *Tridacna squamosa* (Ip et al., 2017), *Mytilus galloprovincialis* (Perfetto et al., 2017), and *H. tuberculata* (Le Roy et al., 2012). To date, the identification and biomolecular characterization of CA II isoform from the Pacific abalone have not yet been reported. For the first time, the complete sequence of CA II was cloned from the mantle tissue of *H. discus hannai* and the molecular properties of this protein with its expression profile were determined in this study. An 18-amino-acid NH_2_-terminal signal sequence was found in the CA II isozyme followed by a cleavage site, suggesting that *HdhCA II* might be an extracellular secretory protein (Figure 1). The N-terminal signal sequence is a key characteristic of CA secretory protein (Aldred et al., 1991). A secretory CA has been cloned from the scleractinian coral, *Stylophora pistillata*, and this CA is localized in calicodermis, which is responsible for the precipitation of the skeleton (Moya et al., 2008). One CA isoform was isolated from the sea urchin embryo and described as an extracellular secreted protein (Karakostis et al., 2016). The cloned sequence of *HdhCA II* also possesses several key features including phosphorylation sites and N-linked glycosylation sites. These phosphorylation sites are crucial for several signal transduction cascades (Ali et al., 2016). Two potential N-linked glycosylation motifs were found in *HdhCA II*, suggesting that *HdhCA II* might be a glycoprotein. Two cysteine residues found in *HdhCA II* might form disulfide link that is crucial for stabilizing its protein structure and regulating biological functions of this protein (Kadokura et al., 2004; Inaba et al., 2006).

The amino acid sequences encoded by *HdhCA II* displayed high identities in the functional site of the CA domain (Figure 2). The molecular structure of *HdhCA II* isozyme contained important functional sites, such as zinc binding ligand, proton shuttling ligand, substrate associated pocket, and Thr-199 loop site, which are known to be involved in the enzymatic activity of this protein (Esbaugh and Tufts, 2006). The active site of CA contained a hydrophobic pocket (i.e., the catalytically productive site) that could interact with a non-polar CO_2_ substrate (Liang and Lipscomb, 1990) and facilitate its reaction with highly nucleophilic Zn^2+^-bound OH^–^ (Alterio et al., 2012). The hydrophobic binding pocket residue and histidine residues that could bind with a catalytic zinc ion were conserved in CA II of *H. discus hannai* (Figure 2). This suggests that *HdhCA II* is a functionally active CA. It has been well established that the proton-shuttling residue (His-64) is responsible for the efficient proton transfer and the high catalytic rate of CO_2_ hydration in CA II of human (Supuran, 2008). A site-specific mutation (His-64 replaced by alanine) results in 20- to 30-fold decrease in the catalytic activity of CA II (Tu et al., 1989). This residue is also conserved among different species of abalone CA II.

The results of phylogenetic analysis indicated that CA II gene of *H. discus hannai* was evolutionarily closer to *H. gigantea* CA II (Figure 3). Previous studies reported that the CA II of *H. tuberculata* (htCA2) is placed in the molluscan CA clade and more closely linked to CA II isoform of *H. gigantea* (Le Roy et al., 2012).

The homology modeling of *HdhCA II* was performed using the 3D homology structure of human CA II with a resolution of 1.07 Å as template (Figure 4). The Zn^2+^ coordinated with three conserved histidine residues comprise the zinc-binding site (Christianson and Alexander, 1989). The evaluation results also supported the structural conservation of the cloned *HdhCA II* gene, with amino acids in favorable positions.

The expression of *HdhCA II* mRNA was detected in all tested tissues with mantle as the site of highest expression (Figure 5) which is in agreement with the previous report (Le Roy et al., 2012; Ip et al., 2017). The expression analysis suggests that *HdhCA II* could involve in mantle function such as shell formation. *HdhCA II* might also involve in acid–base regulation, ion transport, and modulation of ionic concentration (Miyashita et al., 2012).

The temporal expression profile of *HdhCA II* during ontogenesis revealed that *HdhCA II* mRNA was expressed throughout the early developmental stages, with shell formation stage having the highest level (Figure 6). This result of analysis suggests that *HdhCA II* plays an important role during larval shell formation. Previous studies have reported that the functional inhibition of CA in *Paracentrotus lividus* and *Heliocidaris tuberculata* can prevent the deposition of calcium carbonate in the larval skeleton formation (Zito et al., 2015).

The expression of *HdhCA II* mRNA in the mantle tissue was examined with ISH using an antisense CA II mRNA as a probe. The gene distributed in the mantle can speculate the participation of these genes in the biomineralization process during shell formation (Suzuki and Nagasawa, 2013). The expression of genes at the mantle edge and mantle pallial have participated in the synthesis of prismatic and nacreous layers, respectively (Takeuchi and Endo, 2006; Inoue et al., 2010). The expression patterns of *HdhCA II* transcript were detected in the epithelium layer of the mantle and mantle pallial (Figure 5). The mantle can secrete biomineralization protein in outer epithelial cells to modulate shell formation (Jablonski, 1990; Werner et al., 2013). Based on the *in situ* results of the present study, we speculated that *HdhCA II* might be involved in the shell formation by catalyzing the hydration of CO_2_.

## Conclusion

This is the first study of molecular characterization and expression of *HdhCA II* mRNA in different tissues and developmental stages of the Pacific abalone. *HdhCA II* was highly expressed in the mantle tissue implying that it might be participated in the shell formation process. The expression patterns of *HdhCA II* during larval developmental stages imply that this enzyme is involved in the shell germination of abalone. The ISH results demonstrated that the signals were found in the mantle epithelial cells, indicating that this gene might be essential for the shell formation by controlling the regular deposition of CaCO_3_. The findings of our current research could help us to understand the functional role of CA in the shell formation of abalone or be useful for the development of aquaculture methods in this abalone species.

## Data Availability Statement

The original contributions presented in the study are publicly available. This data can be found here: NCBI GenBank, accession number: MT876410, https://www.ncbi.nlm.nih.gov/nuccore/MT876410.1.

## Ethics Statement

The animal study was reviewed and approved by the Institutional Animal Care and Use Committee of CNU (approval number: CNU IACUC-YS-2020-5).

## Author Contributions

KK conceptualized and designed the experiments and prepared the manuscript. MS designed and conducted the experiment, analyzed the data, and wrote the manuscript. ZS conducted the ISH, analyzed the data, and prepared graphs. KS analyzed the data. SC and KC helped to plan the experiment, revised the manuscript, and gave intellectual input to improve it. All authors read and approved the final manuscript.

## Conflict of Interest

The authors declare that the research was conducted in the absence of any commercial or financial relationships that could be construed as a potential conflict of interest.

## Footnotes

1 https://salilab.org/modeller/

## Supplementary Material

Supplementary Figure 1 *HdhCA II* mRNA expression in the cerebral ganglion, shell muscle, mantle, gill, heart, hemocyte, testis, and ovary was determined by semiquantitative reverse transcription (RT)-PCR. RPL-5 was used as reference gene.
