# Supplementary figures and images for: Molecular Characterization of Carbonic Anhydrase II (CA II) and Its Potential Involvement in Regulating Shell Formation in the Pacific Abalone, Haliotis discus hannai
Source: Front Mol Biosci. 2021 May 7;8:669235. doi: 10.3389/fmolb.2021.669235 (PMC8138131; doi:10.3389/fmolb.2021.669235)

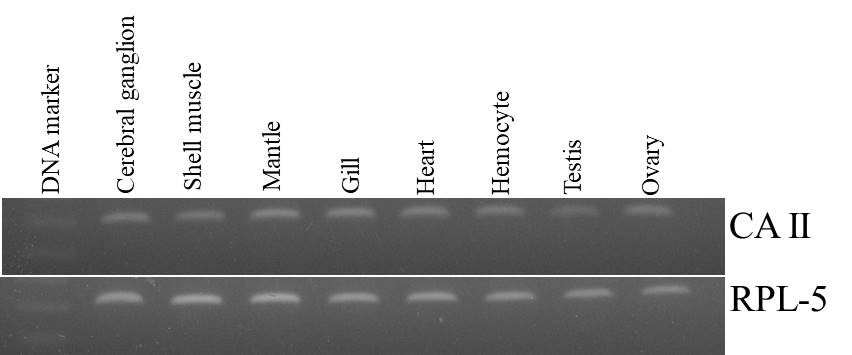

Supplement: Supplementary Figure 1 — HdhCA II mRNA expression in the cerebral ganglion, shell muscle, mantle, gill, heart, hemocyte, testis, and ovary was determined by semiquantitative reverse transcription (RT)-PCR. RPL-5 was used as reference gene. [file Image_1.JPEG]
